# Agricultural Work as a Cure for the Sequels of War Wounds

**Published:** 1919-01

**Authors:** 


### ORTHOPEDICS

**Agricultural Work as a Cure for the Sequels of War Wounds.** By J. Bergonie. *Bulletin de l'Académie de Médecine,* July 9, 1918.

What follows is the result of more than three years' experience during which the writer has been led more and more to substitute agricultural employment for mechanotherapy and even for physiotherapy.

This is a new, natural physiotherapy more efficacious than the old one, at any rate where war wounds are concerned, and when the subjects are young and healthy at the time of the wounds.

It is already very well known that the two chief factors which hasten the cure of wounds are time and functional activity, — the latter, of course, under the guidance of a doctor. The writer believes that one could not get a higher percentage of work in a minimum of time than by sending a wounded man to work all day and every day in the fields according to the formula of the agricultural cure. The quality as well as the quantity of functional activity are superior here to whatever has been done in hospitals, when wounded men are shut in to undergo artificial physiotherapy, without mentioning the physical and moral environment which is much more favorable.

There are incapacities, the exact form of which is indefinite, and which are constituted by a variety of causes.

Here is a man, for instance, who can not completely stretch his arm because the triceps is not sufficient, and his articulation has no longer the required surface of revolution, and also because he does not now know exactly how to command his movement.

The writer does not deny that a well-thought-out apparatus could, in the hands of a doctor who would give the necessary time to each wounded man, produce very encouraging results. But this is peace-time treatment. With the number of wounded whom we have to attend, it is a material impossibility, and it would be impossible to find the specialists who could watch every patient step by step.

The same could be said about electrotherapy (tho for different reasons) and today surgeons, whom the examination of our men, completely cured, has convinced, ask us to take new ones in the fields as rapidly as possible.

Experience has taught us, too, that early dispatoh of the wounded to the land, when the surgeon can do no more, has yielded more than was expected, such as the expulsion of bone splinters or fragments of material which were still keeping the wound suppurating.

It had also to be demonstrated that recently slightly wounded could go to the fields immediately, provided with a dressing easily renewed. They recover better and more quickly than in town hospitals where they do nothing.

The writer does not wish to insist here on what he has previously called the sub-product of such cures; that is, the considerable help given to agriculture by the wounded in the Gironde department, or the 18th region. Aside from the far superior results obtained in the agricultural hospital, 10700 days of work in a year are not a negligible point.

